# Risk factors for medical device-related pressure injury in ICU patients: A systematic review and meta-analysis

**DOI:** 10.1371/journal.pone.0287326

**Published:** 2023-06-23

**Authors:** Ling Gou, Zhiqin Zhang, Yongde A.

**Affiliations:** 1 Department of Gastrointestinal surgery, Xining, China; 2 Intensive Care Unit, Qinghai Provincial People’s Hospital, Xining, China; Children’s National Hospital, George Washington University, UNITED STATES

## Abstract

**Background:**

Medical device-related pressure injury (MDRPI) in intensive care unit (ICU) patients is a serious issue. We aimed to evaluate the risk factors for MDRPI associated with ICU patients through systematic review and meta-analysis, and provide insights into the clinical prevention of MDRPI.

**Methods:**

We searched PubMed, Embase, Web of Science, China National Knowledge Infrastructure (CNKI), WanFang Database, and China BioMedical Literature Database (CBM) (from inception to January 2023) for studies that identified risk factors of MDRPI in ICU patients. In order to avoid the omission of relevant literature, we performed a secondary search of the above database on February 15, 2023. Meta-analysis was performed using Revman 5.3.

**Results:**

Fifteen studies involving 4850 participants were selected to analyze risk factors for MDRPI in ICU patients. While conducting a meta-analysis, we used sensitivity analysis to ensure the reliability of the results for cases with significant heterogeneity among studies. When the source of heterogeneity cannot be determined, we only described the risk factor. The risk factors for MDRPI in ICU patients were elder age (OR = 1.06, 95% CI: 1.03–1.10), diabetes mellitus (OR = 3.20, 95% CI: 1.96–5.21), edema (OR = 3.62, 95% CI: 2.31–5.67), lower Braden scale score (OR = 1.22, 95%CI: 1.11–1.33), higher SOFA score (OR = 4.21, 95%CI: 2.38–7.47), higher APACHE II score (OR = 1.38, 95%CI: 1.15–1.64), longer usage time of medical devices (OR = 1.11, 95%CI: 1.05–1.19), use of vasoconstrictors (OR = 6.07, 95%CI: 3.15–11.69), surgery (OR = 4.36, 95% CI: 2.07–9.15), prone position (OR = 24.71, 95% CI: 7.34–83.15), and prone position ventilation (OR = 17.51, 95% CI: 5.86–52.36). Furthermore, we found that ICU patients who used subglottic suction catheters had a higher risk of MDRPI, whereas ICU patients with higher hemoglobin and serum albumin levels had a lower risk of MDRPI.

**Conclusion:**

This study reported the risk factors for MDRPI in ICU patients. A comprehensive analysis of these risk factors will help to prevent and optimize interventions, thereby minimizing the occurrence of MDRPI.

## Background

Pressure injury (PI) is a local injury to the skin and/or subcutaneous tissue caused by pressure or a combination of shear forces. It usually occurs at bone protuberances, but may also be associated with medical devices or other objects [1]. Patients suffering from PI may experience psychological issues such as social isolation and an inferiority complex [2]. It is also related to poor prognosis of patients, such as decreased quality of life, prolonged hospitalization, increased infection rate, increased readmission rate, hospital mortality, and significantly increased mortality within 30 days of discharge [3, 4]. The direct medical costs associated with PI, as well as the indirect medical and non-medical costs of prolonged hospitalization, productivity loss, and health life year loss are also significant [5, 6]. Pressure injury has emerged as a major public health concern. Updating the concept of PI by organizations such as National Pressure Ulcer Advisory Panel (NPUAP) emphasizes the importance of MDRPI in the clinic. Also, with the advancement of medical technology and the increase in the use rate of medical devices, the possibility of patients suffering from medical device-related pressure injury (MDRPI) has increased [7], and the incidence of iatrogenic injury caused by MDRPI has also shown an upward trend [8]. Researchers have begun to pay attention to the problems related to MDRPI. The term MDRPI refers to the pressure injury caused by medical devices for diagnosis or treatment. It is an important part of hospital-acquired pressure injury (HAPI) [9]. The shape of the injury site is usually consistent with the shape of the medical device [10]. It includes skin medical device-related pressure injury (MDR-S PI) and mucous membrane medical device-related pressure injury (MDR-MM PI) [11]. Among them, MDR-MM PI is the local mucosal injury caused using medical devices, a distinct type of MDRPI. Because of the histological differences between mucosa and skin, we cannot use the PI staging system of the NPUAP based on skin anatomical structure to stage the PI of the mucosa [12, 13].

ICU patients require more medical equipment or instruments due to the need for treatment and disease monitoring. They will have more opportunities to use certain specialized devices or certain specific drugs, making them more susceptible to MDRPI [14, 15]. Furthermore, compared with patients in the general ward, ICU patients are more likely to have problems such as limited activity, perception, and disturbance of consciousness, leading to MDRPI in patients [16]. Cao *et al*. [17] found that the ICU has a high incidence and prevalence of adult MDRPI from a meta-analysis of 21 articles. A study [18] from Australia showed that the overall incidence of MDRPI can reach 27.9%, with 68% occurring in the ICU. Hanonu *et al*. [19] showed that medical devices are responsible for 72.2% of stress injuries in ICUs. Similarly, Black *et al*. [9] found that 34.5% of HAPI in ICUs is related to medical devices. A study by Celik *et al*. [20] involving 302 ICU patients showed that 27.2% had MDRPI. In a prospective study involving 175 ICU patients, Hanonu *et al*. [19] showed that 40.0% had MDRPI. According to a systematic review [21], the incidence rate of MDRPI in ICU patients is 0.9%~41.2%, and the prevalence rate is 1.4%~121%. The incidence/prevalence of MDRPI in ICU patients reported by various researchers varies due to the different types of ICU included in the study, different types and quantities of medical devices used, different stages of PI in the study, and other factors. However, it is found that the incidence/prevalence of MDRPI in ICU patients is relatively high, which has become an important public health issue.

Finding risk factors helps predict and prevent MDRPI in ICU patients for clinical practice. Researchers found many potential risk factors associated with MDRPI in ICU patients, but the risk factors for MDRPI reported in different studies vary. Therefore, we conducted a systematic review and meta-analysis to clarify the risk factors for MDRPI in ICU patients, expecting to provide a scientific foundation for reducing the incidence of MDRPI in ICU patients.

## Methods

### Search strategy

We searched PubMed, Embase, Web of Science, China National Knowledge Infrastructure (CNKI), WanFang Database, and China Biomedical Literature Database (CBM) (from inception to January 2023) for studies that identified risk factors of MDRPI in ICU patients. During the search, we used terms such as stress injury, pressure ulcer, and pressure injury to improve its comprehensiveness, but not limited to device-related terms. Instead, we read the full text to clarify whether the study explores the risk factors for MDRPI. The search formula used in this study is: ("pressure ulcer" [Mesh] OR "pressure ulcer" [Title/Abstract] OR "pressure injury" [Title/Abstract] OR "stress ulcer" [Title/Abstract] OR "stress injury" [Title/Abstract]) AND ("intensive care units" [Mesh] OR "intensive care units" [Title/Abstract] OR "ICU" [Title/Abstract]).

The language used in the search was not restricted. For the literature search, we combined subject words and free words. Simultaneously, we conducted a supplementary search using the snowball method. In order to avoid the omission of relevant literature, we performed a secondary search of the above database on February 15, 2023.

### Inclusion and exclusion criteria

The inclusion criteria: (1) Published case-control studies, cohort studies, and cross-sectional studies; (2) Participants were ≥18 years old, and the occurrence of PI was related to the medical device used; (3) Relevant data can be obtained, either directly or after calculation; (3) The OR (95%) of risk factors was reported or can be calculated.

The exclusion criteria: (1) Meeting abstracts, review papers, case reports, qualitative studies, and letters; (2) Unable to obtain the full text; (3) Unable to get the data required for meta-analysis; (4) Duplicate publications; (5) Animal experiments; (6) Evaluation of research with low literature quality.

### Methodological quality assessment

The quality of a cohort study and case-control study was evaluated using the Newcastle-Ottawa scale (NOS), which included selection (4 items, a total of 4 points), comparability (1 item, a total of 2 points), exposure/outcome evaluation (3 items, a total of 3 points). The total score on the scale was 9. When the total score was ≤ 3, the study was rated as low quality, 4–6 as medium quality, and ≥ 7 as high quality [22]. The cross-sectional study was evaluated according to the quality evaluation criteria recommended by the Agency for Healthcare Research and Quality (AHRQ), including 11 items. Each item can be answered by "yes" (score 1), "no" (score 0), or "unclear" (score 0). The higher the score, the higher the quality. The total score ≤3 points rated as low quality, 4–7 points as medium quality, and ≥ 8 points as high quality [23].

### Data extraction

Two researchers conducted literature screening and data extraction respectively. The items for data extraction mainly included first authors, publication year, study design, type of MDRPI, sociodemographic data, sample size, risk factors, detailed information on methodology, etc. In case of disagreement, it should be resolved through discussion.

### Statistical analysis

Revman 5.3 software was used for meta-analysis. The heterogeneity test among studies was examined using Cochran’s Q (χ^2^) test and I^2^ statistics. When *P* > 0.1 and I^2^ < 50%, the fixed effect model was used for meta-analysis. For *P* < 0.1 and I^2^ ≥ 50%, sensitivity analysis was carried out by subgroup analysis, changing the effect model or leave-one-out method. When the source of heterogeneity could not determine, descriptive analysis was used. The results of pooled OR (95%CI) were illustrated using forest plots. A funnel plot was used to evaluate publication bias. Statistical significance was set at *P* value < 0.05.

### Patient and public involvement

No patient was involved in this study.

## Results

### Literature selection

**Fig 1:**
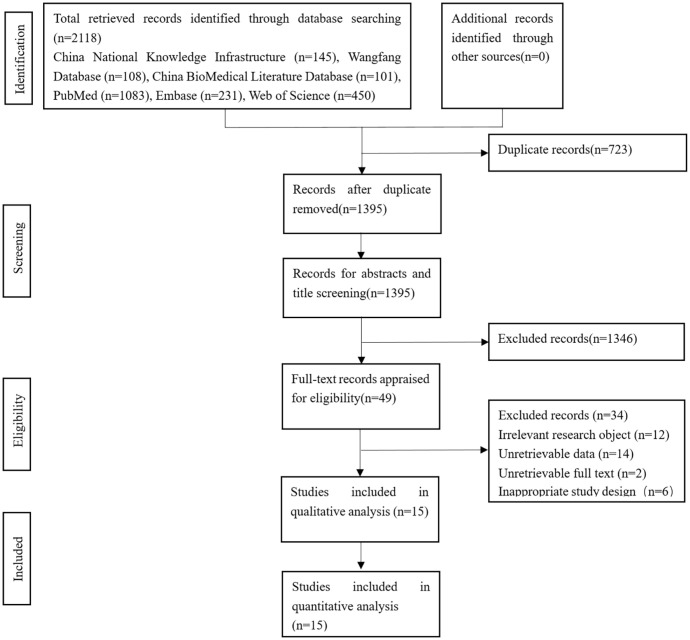
Flow chart.

During the initial examination, a total of 2,118 studies were obtained, and 723 studies were deleted by endnote. According to the inclusion and exclusion criteria, 1346 studies were deleted after reading the title and abstract, and 0 were included in other ways. After reading the full text, 15 studies [19, 24–37] with 4,850 participants were finally included (Fig 1).

### Study characteristics

The included studies were conducted in three countries: China (n = 12), Korea (n = 2), and Turkey (n = 1). The cohort study included eight articles of medium or high quality. The case-control study included one high-quality article. The cross-sectional study consisted of six articles of medium quality. A total of 4,850 subjects were included in the study, with a sample size ranging from 156 to 912. The average/median age of the included participants ranged from 53.88 to 68.1 years. Table 1 depicts additional information.

**Table 1:** Basic information of included articles.

Author, year	Country	Sample size (n)	Age ($\bar{\text{χ}}\pm S$)/ M(P25, P75)	Type of medical device	Type of MDRPI	Study design	NOS/AHRQ scores
Choi BK 2020 [24]	Korea	194	63.19±11.83	endotracheal tube	MDR-MM PI	cohort study	6
Dang W 2022 [25]	China	694	65.0±17.4	unrestricted types of medical devices	MDRPI	cross-sectional study	6
Dong ZH 2023 [37]	China	280	55.55±12.26	unrestricted types of medical devices	MDRPI	cross-sectional study	5
Hanonu S 2016 [19]	Turkey	175	62.50±16.67	unrestricted types of medical devices	MDRPI	cohort study	7
He LY 2020 [29]	China	189	-	nasal mask	MDRPI	cohort study	6
Koo M 2019 [26]	Korea	253	-	Unrestricted types of medical devices	MDRPI	cohort study	7
Liu D 2022 [30]	China	158	64.0(50.0, 71.0)	endotracheal intubation	MDR-MM PI	cohort study	6
Nan RL 2023 [27]	China	912	53.88±17.70	indwelling transnasal tubes	MDR-MM PI	cross-sectional study	7
Qi JF 2022a [31]	China	210	-	unrestricted types of medical devices	MDRPI	cross-sectional study	6
Qi JF 2022b [32]	China	280	-	unrestricted types of medical devices	MDRPI	cross-sectional study	6
Qin LL 2020 [33]	China	156	68.1±14.9	endotracheal intubation	MDRPI	case-control study	7
Wang J 2015 [34]	China	299	57.8±16.9	unrestricted types of medical devices	MDRPI	cross-sectional study	4
Wu D 2020 [35]	China	181	61.34±14.03	medical devices for oxygen therapy	MDRPI	cohort study	5
Zhang YB 2021 [28]	China	219	55.40±15.15	nasogastric tube	MDR-MM PI	cohort study	7
Zhou XL 2022 [36]	China	650	67(55,76)	artificial airway	MDRPI	cohort study	7

Notes: -, not reported. MDR-MM PI, mucous membrane medical device-related pressure injury. MDRPI, medical device-related pressure injury.

### Results of meta-analysis

#### Demographic data

**Fig 2:**
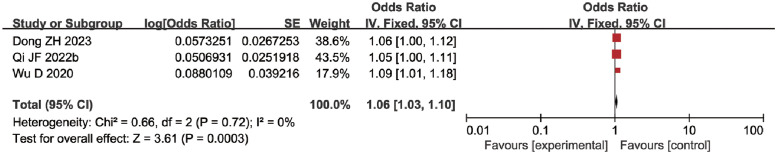
Meta-analyses for the association between age and MDRPI.

*Age*. Four studies [29, 32, 35, 37] have reported the impact of age on MDRPI in ICU patients. Significant heterogeneity exists among studies (*P* = 0.002, I^2^ = 84%). After removing each included study one by one, it was found that the study by He *et al*. [29] was the primary source of heterogeneity. After excluding this study, there was no significant heterogeneity among the studies (*P* = 0.42, I^2^ = 0%). A meta-analysis using a fixed effect model showed that older patients had a higher risk of MDRPI [OR = 1.06, 95%CI (1.03, 1.10), *P* = 0.0003] (Fig 2).

#### Disease data

**Fig 3:**
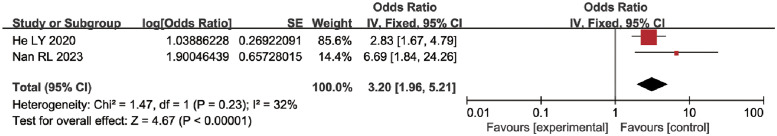
Meta-analyses for the association between diabetes and MDRPI.

*Diabetes*. Two studies [27, 29] reported the impact of diabetes on MDRPI in ICU patients. The heterogeneity between studies was small (*P* = 0.23, I^2^ = 32%). A meta-analysis using a fixed effect model showed that ICU patients with diabetes were more likely to develop MDRPI [OR = 3.20, 95%CI (1.96, 5.21), *P* < 0.00001] (Fig 3).

*Hemoglobin*. Two studies [32, 37] have reported the effect of hemoglobin on MDRPI in ICU patients. Significant heterogeneity exists between studies (*P* = 0.05, I^2^ = 75%), and changing the effect model revealed substantial differences in results. Therefore, only descriptive analysis was conducted. Both studies showed that higher hemoglobin levels are protective factors against MDRPI in ICU patients.

*Serum albumin*. Three studies [24, 27, 28] have reported the effect of serum albumin on MDRPI in ICU patients. There is significant heterogeneity among studies (*P* < 0.0001, I^2^ = 91%), and sensitivity analysis cannot determine the source of heterogeneity. Thus, only descriptive analysis was conducted. All three studies showed that the higher the serum albumin level, the lower the risk of MDRPI in ICU patients.

**Fig 4:**
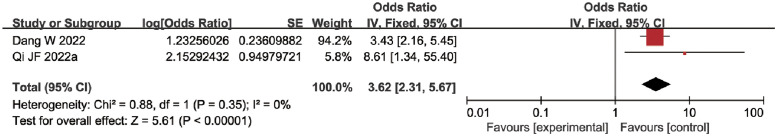
Meta-analyses for the association between edema and MDRPI.

*Edema* Two studies [25, 31] have reported the impact of edema on MDRPI in ICU patients. There was no significant heterogeneity between studies (*P* = 0.35, I^2^ = 0%). ICU patients with edema were found to have a higher risk of MDRPI based on meta-analysis using a fixed effect model [OR = 3.62, 95%CI (2.31, 5.67), *P* < 0.00001] (Fig 4).

**Fig 5:**
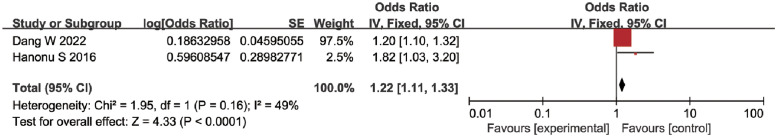
Meta-analyses for the association between Braden scale score and MDRPI.

*Braden scale score*. Four studies [19, 25, 33, 36] have reported the impact of the Braden scale score on MDRPI in ICU patients. Two studies [19, 25] examined the impact of the total score of the Braden scale score on the occurrence of MDRPI in ICU patients. There is little heterogeneity between studies (*P* = 0.16, I^2^ = 49%). Meta-analysis using a fixed effect model showed that the lower the total Braden scale score, the greater the risk of MDRPI in ICU patients [OR = 1.22, 95%CI (1.11, 1.33), *P* < 0.0001] (Fig 5). Two studies [33, 36] have reported the relationship between the Braden scale score for moisture, mobility, friction, shear, and MDRPI in ICU patients. However, there was significant heterogeneity between these two studies while discussing the impact of moisture score on MDRPI in ICU patients (*P* < 0.00001, I^2^ = 97%), and only descriptive analysis was used. Qin *et al*. [33] found that a lower moisture score during intubation is a protective factor for MDRPI in ICU patients, whereas Zhou [36] showed that a lower moisture score is a risk factor for MDRPI in ICU patients. However, significant heterogeneity was observed between the above two studies while discussing the impact of the mobility score on MDRPI in ICU patients (*P* = 0.06, I^2^ = 72%). The risk of MDRPI in ICU patients was found to be inversely correlated with mobility score during intubation using a randomized effect model for meta-analysis [OR = 3.13, 95%CI (1.48, 6.63), *P* = 0.003]. There was significant heterogeneity between the studies of Zhou [36] and Qin *et al*. [33] when discussing the impact of friction and shear score on MDRPI in ICU patients (*P* = 0.14, I^2^ = 53%). A meta-analysis using a random effect model showed that the lower the score of friction and shear during intubation, the higher the risk of MDRPI in ICU patients [OR = 4.26, 95%CI (1.98, 9.18), *P* = 0.0002].

**Fig 6:**
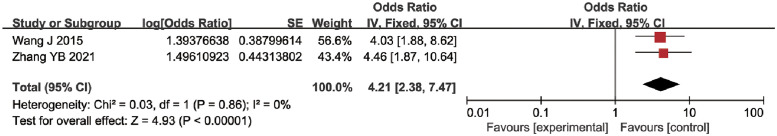
Meta-analyses for the association between SOFA score and MDRPI.

*Sequential organ failure assessment (SOFA)*. Two studies [28, 34] examined the impact of SOFA score on MDRPI in ICU patients. There was no significant heterogeneity between studies (*P* = 0.86, I^2^ = 0%). A meta-analysis using a fixed effect model showed that the higher the SOFA score, the greater the risk of MDRPI in ICU patients [OR = 4.21, 95% CI (2.38, 7.47), *P* < 0.00001] (Fig 6).

**Fig 7:**
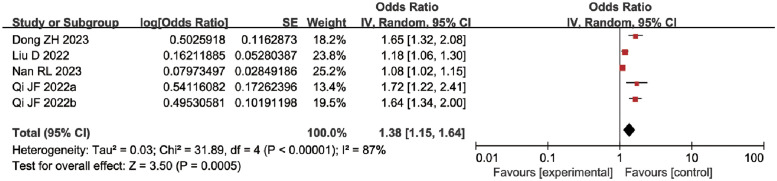
Meta-analyses for the association between APACHE II score and MDRPI.

*APACHE II score*. The impact of the APACHE II score on MDRPI in ICU patients has been reported in five studies [27, 30–32, 37]. There is significant heterogeneity between studies and no significant difference in results when the effect model is changed (*P* < 0.00001, I^2^ = 87%). Thus, a meta-analysis using a random effect model shows that the higher the APACHE II score, the greater the risk of MDRPI in ICU patients [OR = 1.38, 95%CI (1.15, 1.64), *P* = 0.0005] (Fig 7).

#### Device factors

**Fig 8:**
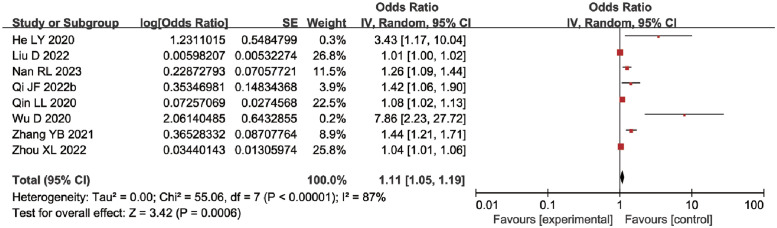
Meta-analyses for the association between usage time of medical devices and MDRPI.

*Usage time of medical devices*. Eight studies [27–30, 32, 33, 35, 36] have reported the impact of medical device usage time on MDRPI in ICU patients. The heterogeneity among studies was significant (*P* < 0.00001, I^2^ = 87%), confirmed by subgroup analysis and the leave-one-out method. After changing the effect model, no significant difference was observed in the pooled OR results. A meta-analysis using a random effect model showed that the longer the medical device was used, the higher the risk of MDRPI in ICU patients [OR = 1.11, 95%CI (1.05, 1.19), *P* = 0.0006] (Fig 8).

*The use of a subglottic suction catheter*. Two studies [33, 36] reported the impact of using subglottic suction catheters on MDRPI in ICU patients. Significant heterogeneity existed between studies (*P* = 0.009, I^2^ = 85%). After changing the effect model, the results of the meta-analysis have significantly changed; thus, only descriptive analysis has been conducted. Both studies found that ICU patients who used subglottic suction catheters had a higher risk of MDRPI.

#### Treatment factors

**Fig 9:**
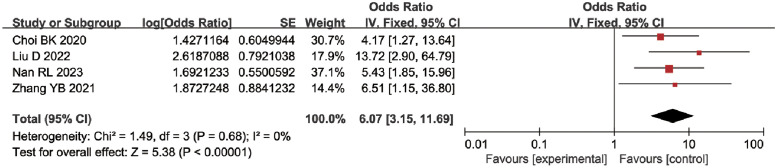
Meta-analyses for the association between the use of vasoconstrictors and MDRPI.

*The use of vasoconstrictors*. Five studies [24, 27, 28, 30, 31] have reported the impact of vasoconstrictor use on medical device-related stress injury in ICU patients. There is significant heterogeneity between studies (*P* = 0.0009, I^2^ = 79%). After removing each of the included studies, it was found that the study of Qi *et al*. ^a^ [31] is the main source of heterogeneity. And there was no significant heterogeneity among the remaining studies (*P* = 0.68, I^2^ = 0%). A meta-analysis using a fixed effect model showed that ICU patients using vasoconstrictors had a greater risk of MDRPI [OR = 6.07, 95%CI (3.15, 11.69), *P* < 0.00001] (Fig 9).

**Fig 10:**
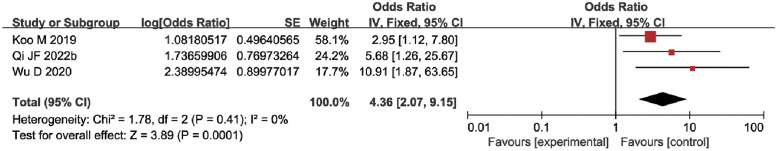
Meta-analyses for the association between surgery and MDRPI.

*Surgery*. Three studies [26, 32, 35] have reported the impact of surgery on MDRPI in ICU patients. There was no significant heterogeneity between studies (*P* = 0.41, I^2^ = 0%). Based on a meta-analysis using a fixed effect model, surgery significantly increased the risk of MDRPI in ICU patients [OR = 4.36, 95%CI (2.07, 9.15), *P* = 0.0001] (Fig 10).

**Fig 11:**
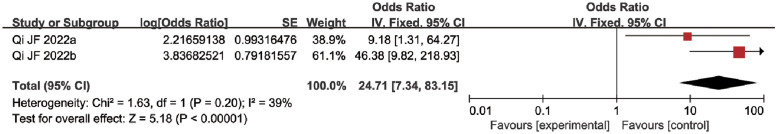
Meta-analyses for the association between position and MDRPI.

*Position*. Two studies [31, 32] have reported the impact of position on MDRPI in ICU patients, where the heterogeneity between them was small (*P* = 0.20, I^2^ = 39%). A meta-analysis using a fixed effect model showed that ICU patients in prone positions had a higher risk of MDRPI [OR = 24.71, 95%CI (7.34, 83.15), *P* < 0.00001] (Fig 11).

**Fig 12:**
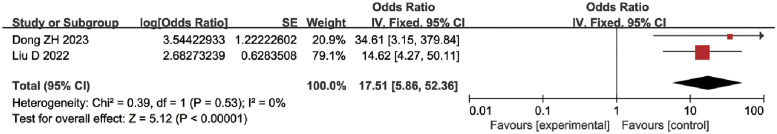
Meta-analyses for the association between prone position ventilation and MDRPI.

*Prone position ventilation*. The effects of prone position ventilation on MDRPI in ICU patients have been reported in two studies [30, 37], where no significant heterogeneity was observed between them (*P* = 0.53, I^2^ = 0%). A meta-analysis using a fixed effect model showed that ICU patients using prone ventilation had a greater risk of MDRPI [OR = 17.51, 95%CI (5.86, 52.36), *P* < 0.00001] (Fig 12).

#### Other risk factors

There were 13 risk factors in the study that were eligible for meta-analysis and evaluation. Other risk factors are reported based on the results of individual studies. Among them, patients with traditional HAPI have a 6.6 times higher risk of MDRPI than others. The risk of MDRPI in ICU patients receiving enteral nutrition is 2.12 times higher than that of other patients. The risk of MDRPI in patients in the medical ICU, neurosurgical ICU, and Chest diseases ICU is 7.041, 6.221, and 6.014 times higher than in other ICU patients [19]. According to Koo *et al*. [26], the risk of MDRPI in ICU patients with semi-coma/coma and sedation is 5.79 times and 5.54 times higher than in other ICU patients. Nan [27] showed that ICU patients with disturbance of consciousness have a higher risk of nasal mucosal MDR-MM PI [OR = 4.231, 95%CI (1.668, 10.733), *P* = 0.002]. Table 2 depicts specific information on additional risk factors.

**Table 2:** Specific information on other risk factors of MDRPI in ICU patients.

Risk factors	OR	LL-95%CI	UL-95%CI	P value
Local skin with damp heat [29]	2.756	3.875	33.937	0.001
Fever [27]	3.438	1.400	8.443	0.007
BMI [35]	1.285	1.016	1.625	<0.05
C-reactive protein [28]	0.656	0.506	0.852	0.002
Number of medical devices used [37]	1.915	1.225	2.994	0.004
Use of non-invasive ventilation mask [37]	4.366	1.044	18.258	0.043
Use of endotracheal catheters [26]	5.79	1.66	20.20	0.006
The position of the tracheal tube in the mouth [36]	4.937	1.323	18.427	0.017
ICU stay time [34]	2.061	1.293	3.286	0.002

Notes: BMI, Body mass index. ICU, intensive care unit.

### Publication bias evaluation

No publication bias was evaluated due to the small number of studies on each risk factor in this study.

## Discussion

This article aims to study the risk factors for MDRPI in ICU patients. Eventually, this study included 14 risk factors, of which three were only described. Some of the risk factors included in the study were only reported because they were found in individual studies. This meta-analysis revealed that elder age, diabetes mellitus, edema, lower Braden scale score, higher SOFA score, higher APACHE II score, longer use of medical devices, use of vasoconstrictors, surgery, the prone position, the prone position ventilation, and the use of a subglottic suction catheter were associated with a higher risk of developing MDRPI in ICU patients, whereas ICU patients with higher hemoglobin or serum albumin levels had a relatively lower risk of MDRPI.

### Demographic data

According to some studies [38], while PI can occur in patients of all ages, 70–73% of PI occurs in people over the age of 65. The impact of age on MDRPI is multifaceted. As age increases, he integrity of the skin and the repair ability of histiocytes are also weakened, making it more vulnerable to external forces such as moisture, friction, and trauma and less prone to healing [39]. Furthermore, elderly people are more prone to stress damage due to reduced basal metabolism, blood circulation, and sensory retardation, and are often associated with malnutrition, mobility difficulties, and severe complications [40]. When elderly patients in the ICU require long-term, multiple, and majority uses of medical devices, they have poorer physical conditions and are more susceptible to MDRP.

### Disease data

The risk of MDRPI in ICU patients with diabetes will be significantly increased. Elevated blood sugar levels reduce neutrophil activity and weaken the body’s ability to resist bacteria and foreign bodies, which can cause skin damage [41]. According to Chen *et al*. [42], a continuous increase in blood sugar couldlead to the accumulation of glycation end products and pathophysiological changes in the skin. Conversely, peripheral neuropathy caused by microcirculation disorders in diabetes can result in sensory and motor nerve disorders in patients and reduce their response to external stimuli, thus making the skin vulnerable to pressure, damage, infection, and then PI [43].

Decreased hemoglobin levels and anemia are strongly associated with the development of PI [44]. When the hemoglobin level in the body is too low, the oxygen-carrying capacity of red blood cells decreases, and the tissue becomes hypoxic. At this time, oxygen levels of fibroblasts responsible for tissue healing also decrease, affecting collagen formation and increasing tissue sensitivity by inducing ischemia and necrosis [45]. This is more likely to induce stress damage for ICU patients who require long-term use of multiple medical devices.

Studies by Yang *et al*. [46] have shown a significant negative correlation between albumin levels and the severity of PI. Low albumin levels can lead to changes in colloid osmotic pressure and edema formation, affecting the diffusion of oxygen and nutrients to tissues and eventually leading to hypoxia and cell death [45]. Conversely, lower albumin levels can result in a decline in the body’s immune system and weakened tissue repair ability, leading to PI [47]. Most patients admitted to ICU are critically ill with complex conditions, disordered internal environments, low albumin levels, and edema, which are more common than patients in general wards, and therefore are more prone to MDRPI.

This study shows that the lower the total score of the Braden scale score, the higher the risk of MDRPI in ICU patients. Two researchers [33, 36] analyzed six items in the Braden scale score, but they disagreed on the relationship between moisture score and MDRPI in ICU patients. Qin *et al*. [33] found that a low moisture score in patients undergoing endotracheal intubation was a protective factor for MDRPI. They believe this may be related to the fact that factors related to dampness, such as skin impregnation by feces and secretions, are more easily recognized and dealt with by medical staff. However, this finding contradicts the view of most researchers [48, 49]. Furthermore, the Braden scale score alone may not effectively assess the risk of PI in ICU patients [50, 51]. Some researchers propose modifying or combining the Braden scale score with other methods to predict the risk of PI. Thus, further research [52, 53] into the relationship between PI risk assessment tools and the occurrence of MDRPI in ICU patients is required.

SOFA score and APACHE II score are commonly used clinical condition evaluation tools, which have been proven effective in predicting the prognosis of critically ill patients [54, 55]. The higher the score, the more severe the patient’s condition is and the greater the likelihood of having hemodynamic and metabolic disorders. The poorer the patient’s basic state is, the more opportunities for requiring long-term bed rest and using multiple medical devices for a long time, and the more prone to MDRPI [56].

### Device factors

The longer a medical device is used, the more it compresses the local skin or mucosa, resulting in tissue cell deformation, inflammatory edema, local ischemia, and hypoxia, eventually leading to MDRPI. According to Ackland *et al*. [57], the risk of PI increases by 66% for each additional day of use of medical devices. The more medical devices that are used, the more likely there will be local pressure on the body, increasing the risk of MDRPI. According to Black *et al*. [9], the risk of PI for patients increases by 2.4 times for every increase in the type of medical device used. Therefore, we should reasonably wear medical devices for patients, change the position of the medical device or its fixed device according to the patient’s condition and type of medical device, and adopt methods such as regular relaxation and local protective measures to reduce pressure on a fixed part. In clinical practice, it is necessary to evaluate the patient’s condition in a timely, accurate, and dynamic manner to eliminate or reduce the use of medical devices as soon as possible.

The type of medical device can affect the occurrence of MDRPI in ICU patients. According to foreign data, respiratory devices are the primary cause of MDRPI, accounting for 68% of all MDRPI related to respiratory devices [21]. Erbay *et al*. [58] found that among the 12 types of medical devices that cause PI related to medical devices, the most common are endotracheal tubes, urinary catheters, nasogastric tubes, and non-invasive masks. In a study of 2,240 ICU patients, Liu *et al*. [59] found that the most common devices causing MDRPI were masks and straps, orthotics, T-shoes, endotracheal intubation, and fixation straps. Xu *et al*. [60] investigated 727 ICU patients and found that the main medical devices causing MDRPI were ECG monitoring leads, restraint bands, and oxygen saturation probes. Different studies have reported differences in the main devices that cause MDRPI. However, due to the limited number of studies included, this study only reported the impact of non-invasive ventilation masks and subglottic suction catheters on MDRPI in ICU patients. Thus, further research into the relationship between the types of medical devices and MDRPI in ICU patients is necessary.

### Treatment factors

The results of this study indicate that the use of vasoconstrictors is a risk factor for MDRPI in ICU patients. A systematic review [61] of 26 studies showed that the incidence of PI was 10.9% among people who used vasoconstrictors and 3.5% among people who did not use vasoconstrictors. For critically ill patients, vasoconstrictors almost tripled the risk of PI. Cox [62] highlighted a strong association between the use of vasopressors and the occurrence of PI, identifying vasopressors as a risk factor for PI in the general population. Furthermore, Holt *et al*. [63] found that while the dose of vasopressor may not affect the incidence of HAPI, patients receiving high-dose vasopressor develop PI earlier than in low-dose cohorts.

The results of this study indicate that surgery is a risk factor for MDRPI in ICU patients. The impact of surgery on MDRPI is multifaceted. Many factors can contribute to the occurrence of MDRPI, including the length of surgery, intraoperative posture limitations, intraoperative exposure or temperature loss, intraoperative bleeding, and drug use [64, 65].

Certain special positions may be associated with the occurrence of PI [66]. However, some researchers [39] have indicated that position is not an independent risk factor for developing PI in patients. In this study, only the impact of prone position on MDRPI is discussed due to the limited number of reported documents. This was thought to be due to thin facial skin and changes in the center of gravity during a prone position, increasing facial pressure, leading to increased congestion and edema in this area, thereby increasing the risk of facial skin damage [31].

The impact of prone position ventilation on MDRPI in ICU patients is reflected in multiple aspects. First, the prone position itself is a risk factor for the occurrence of MDRPI, as shown in the previous research results of this article. Second, the pressure from the head caused by gravity and the pressure from the patient’s support surface cause double compression on the respiratory tube and its fixation device, which increases the pressure on local skin and mucosa, leading to the occurrence of MDRPI. Furthermore, the duration of prone position ventilation can also influence the occurrence of MDRPI. However, many studies [67, 68] have shown that the longer the ventilation time in the prone position, the more effective it is at improving gas exchange in patients and, eventually, their mortality. Nevertheless, the longer patients are ventilated in the prone position, the greater their risk of PI [69]. Therefore, when patients have to use prone position ventilation for a long time due to disease or treatment factors, we can minimize the occurrence of MDRPI by standardizing the prone position ventilation operation process and properly using local decompression tools with other measures.

Of course, this study has certain limitations. (1) The number of included literature on some risk factors is small, and the reliability of research results needs to be further improved; (2) Most of the original studies included did not distinguish between MDR-S PI and MDR-MM PI; thus, mixed calculations can only be performed during meta-analysis; (3) Some of the studies included all stages of MDRPI, while others included only one or several stages of MDRPI. However, no subgroup analysis was conducted due to the small number of literature included in this study.

## Conclusions

This study systematically evaluated the risk factors for MDRPI, which included demographic data, disease data, device factors, and treatment factors. The results of this study showed that age, diabetes, hemoglobin, serum albumin, edema, Braden scale score, SOFA score, APACHE II score, usage time of medical devices, use of a subglottic suction catheter, vasoconstrictors, surgery, position, and prone position ventilation might be related to the occurrence of MDRPI in ICU patients. A comprehensive analysis of these risk factors will help to prevent and optimize interventions, thereby minimizing the occurrence of MDRPI. However, there is still a lack of data in this area, and more high-quality research is recommended in the future to verify the relevant results.

## Supporting information

S1 Checklist PRISMA 2020 checklist. (DOCX)

S1 Data (DOCX)
