# Supplementary material for: Risk factors for medical device-related pressure injury in ICU patients: A systematic review and meta-analysis
Source: PLoS One. 2023 Jun 23;18(6):e0287326. doi: 10.1371/journal.pone.0287326 (PMC10289390; doi:10.1371/journal.pone.0287326)
Supplement: S1 Data — (DOCX) [file pone.0287326.s002.docx]

| Risk factors | Study | OR | OR-LL | OR-UL | ln(OR) | ln(OR-LL) | ln(OR-UL) | SE |
| --- | --- | --- | --- | --- | --- | --- | --- | --- |
| Age | Dong ZH 2023 | 1.059 | 1.005 | 1.116 | 0.0573251 | 0.0049875 | 0.1097509 | 0.0267253 |
|  | Qi JF 2022b | 1.052 | 1.002 | 1.106 | 0.0506931 | 0.001998 | 0.1007499 | 0.0251918 |
|  | Wu D 2020 | 1.092 | 1.011 | 1.179 | 0.0880109 | 0.0109399 | 0.1646666 | 0.039216 |
| Diabetes | He LY 2020 | 2.826 | 12.863 | 36.955 | 1.0388623 | 2.554355 | 3.609701 | 0.2692209 |
|  | Nan RL 2023 | 6.689 | 1.894 | 24.909 | 1.9004644 | 0.638691 | 3.2152292 | 0.6572802 |
| Hemoglobin | Dong ZH 2023 | 0.149 | 0.024 | 0.942 | -1.903809 | -3.729701 | -0.05975 | 0.9362121 |
|  | Qi JF 2022b | 0.958 | 0.934 | 0.983 | -0.042908 | -0.068279 | -0.017146 | 0.0130441 |
| Serum albumin | Choi BK 2020 | 0.09 | 0.03 | 0.24 | -2.407946 | -3.506558 | -1.427116 | 0.5304698 |
|  | Nan RL 2023 | 0.809 | 0.74 | 0.884 | -0.211956 | -0.301105 | -0.123298 | 0.0453589 |
|  | Zhang YB 2021 | 0.5555556 | 0.4050223 | 0.7616146 | -0.587787 | -0.903813 | -0.272315 | 0.1610966 |
| Edema | Dang W 2022 | 3.43 | 2.16 | 5.45 | 1.2325603 | 0.7701082 | 1.6956156 | 0.2360988 |
|  | Qin QJ 2022a | 8.61 | 1.338 | 55.389 | 2.1529243 | 0.291176 | 4.014381 | 0.9497972 |
| Braden scale score | Dang W 2022 | 1.2048193 | 1.0989011 | 1.3157895 | 0.1863296 | 0.0943107 | 0.2744368 | 0.0459506 |
|  | Hanonu S 2016 | 1.815 | 1.029 | 3.205 | 0.5960855 | 0.0285875 | 1.1647121 | 0.2898277 |
| SOFA score | Wang J 2015 | 4.03 | 1.8838 | 8.6213 | 1.3937664 | 0.633291 | 2.1542359 | 0.3879961 |
|  | Zhang YB 2021 | 4.4642857 | 1.8726592 | 10.638298 | 1.4961092 | 0.6273594 | 2.3644605 | 0.443138 |
| APACHE Ⅱ score | Dong ZH 2023 | 1.653 | 1.316 | 2.076 | 0.5025918 | 0.2745968 | 0.730443 | 0.1162873 |
|  | Liu D 2022 | 1.176 | 1.061 | 1.305 | 0.1621188 | 0.0592119 | 0.266203 | 0.0528039 |
|  | Nan RL 2023 | 1.083 | 1.024 | 1.145 | 0.079735 | 0.0237165 | 0.1354046 | 0.0284919 |
|  | Qi JF 2022a | 1.718 | 1.225 | 2.41 | 0.5411608 | 0.2029408 | 0.8796267 | 0.172624 |
|  | Qi JF 2022b | 1.641 | 1.344 | 2.004 | 0.4953058 | 0.2956502 | 0.6951452 | 0.101912 |
| usage time of medical devices | He LY 2020 | 3.425 | 3.368 | 28.915 | 1.2311015 | 1.2143191 | 3.3643605 | 0.5484799 |
|  | Liu D 2022 | 1.006 | 0.996 | 1.017 | 0.0059821 | -0.004008 | 0.0168571 | 0.0053227 |
|  | Nan RL 2023 | 1.257 | 1.095 | 1.444 | 0.2287279 | 0.0907544 | 0.367417 | 0.0705772 |
|  | Qin QJ 2022b | 1.424 | 1.065 | 1.905 | 0.3534698 | 0.0629748 | 0.644482 | 0.1483437 |
|  | Qin LL 2020 | 1.0752688 | 1.0204082 | 1.1363636 | 0.0725707 | 0.0202027 | 0.1278334 | 0.0274568 |
|  | Wu D 2020 | 7.857 | 2.227 | 27.725 | 2.0614049 | 0.8006554 | 3.3223345 | 0.6432855 |
|  | Zhang YB 2021 | 1.4409222 | 1.2150668 | 1.7094017 | 0.3652833 | 0.1947991 | 0.5361434 | 0.0870776 |
|  | Zhou XL 2022 | 1.035 | 1.009 | 1.062 | 0.0344014 | 0.0089597 | 0.0601539 | 0.0130597 |
| The use of a subglottic suction catheter | Qin LL 2020 | 1.4 | 1.1 | 1.78 | 0.3364722 | 0.0953102 | 0.5766134 | 0.1227814 |
|  | Zhou XL 2022 | 2.878 | 1.773 | 4.67 | 1.0570956 | 0.572673 | 1.5411591 | 0.2470628 |
| the use of vasoconstrictors | Choi BK 2020 | 4.1666667 | 1.3333333 | 14.285714 | 1.4271164 | 0.2876821 | 2.65926 | 0.6049944 |
|  | Liu D 2022 | 13.718 | 2.904 | 64.789 | 2.6187088 | 1.0660891 | 4.1711358 | 0.7921038 |
|  | Nan RL 2023 | 5.431 | 1.848 | 15.964 | 1.6921233 | 0.614104 | 2.7703362 | 0.5500592 |
|  | Zhang YB 2021 | 6.506 | 1.15 | 36.801 | 1.8727248 | 0.1397619 | 3.605525 | 0.8841232 |
| Surgery | Koo M 2019 | 2.95 | 1.11 | 7.77 | 1.0818052 | 0.10436 | 2.0502702 | 0.4964057 |
|  | Qi JF 2022b | 5.678 | 1.256 | 25.669 | 1.7365991 | 0.2279321 | 3.245284 | 0.7697326 |
|  | Wu D 2020 | 10.913 | 1.871 | 63.661 | 2.3899547 | 0.626473 | 4.1535721 | 0.8997702 |
| position | Qin QJ 2022a | 9.176 | 1.31 | 64.279 | 2.2165914 | 0.2700271 | 4.163233 | 0.9931648 |
|  | Qin QJ 2022b | 46.378 | 7.784 | 173.467 | 3.8368252 | 2.0520703 | 5.1559874 | 0.7918156 |
| prone position ventilation | Dong ZH 2023 | 34.613 | 3.154 | 379.858 | 3.5442293 | 1.1486715 | 5.9397975 | 1.222226 |
|  | Liu D 2022 | 14.625 | 4.268 | 50.113 | 2.6827324 | 1.4511453 | 3.9142805 | 0.6283508 |
